# Untargeted metabolomics of saliva in caries-active and caries-free children in the mixed dentition

**DOI:** 10.3389/fcimb.2023.1104295

**Published:** 2023-04-04

**Authors:** Yueheng Li, Zhengyan Yang, Ting Cai, Dan Jiang, Jun Luo, Zhi Zhou

**Affiliations:** ^1^ Department of Preventive Dentistry, Stomatological Hospital of Chongqing Medical University, Chongqing, China; ^2^ College of Stomatology, Chongqing Medical University, Chongqing, China; ^3^ Chongqing Key Laboratory of Oral Diseases and Biomedical Sciences, Chongqing, China; ^4^ Chongqing Municipal Key Laboratory of Oral Biomedical Engineering of Higher Education, Chongqing, China

**Keywords:** caries, saliva, untargeted metabolomics, children, mixed dentition

## Abstract

**Objective:**

To compare the differences in salivary metabolites between caries-active and caries-free children in the mixed dentition, and explore their correlation with caries status.

**Methods:**

The study involved 20 children (aged 8–9 years) in the mixed dentition, including 10 caries-active (aged 8.6 ± 0.49years) and 10 caries-free children(aged 8.5 ± 0.5years), with a male/female ratio of 1:1. The saliva samples were collected from all children. Metabolite extraction, LC-MS/MS-based untargeted metabolomics, qualitative and semi-quantitative analysis and bioinformatics analysis were performed to identify differential metabolites between the two sample groups. The differential metabolites identified were further analyzed in an attempt to find their correlations with caries status.

**Results:**

In the positive ion mode, a total of 1606 molecular features were detected in the samples of the two groups, 189 of which were differential metabolites when comparing the caries-active group with the caries-free group, including 104 up-regulated and 85 down-regulated metabolites. In the negative ion mode, a total of 532 molecular features were detected in the samples of two groups, 70 of which were differential metabolites when comparing the caries-active group with the caries-free group, including 37 up-regulated and 33 down-regulated metabolites. In the positive ion mode, two of the top 5 up-regulated differential metabolites were found in and annotated to specific metabolic pathways, whereas in the negative ion mode, only one of the top 5 up-regulated differential metabolites was found in and annotated to specific metabolic pathways. In both the positive and negative ion modes, the top 5 down-regulated differential metabolites were both annotated to the metabolic pathways. KEGG pathway enrichment analysis of differential metabolites showed that histamine and arachidonic acid identified in the positive ion mode, as well as succinate and L-histidine identified in the negative ion mode were enriched in the top 3 significantly altered pathways.

**Conclusion:**

The enriched differential metabolites including histamine, L-histidine and succinate were correlated with the presence of dental caries, but their role in the caries process needs to be further investigated.

## 1 Introduction

Caries is the most common chronic disease that endangers oral health, and is a biofilm-mediated, diet modulated, multifactorial, non-communicable, dynamic disease resulting in net mineral loss of dental hard tissues. Saliva is a mixed fluid that plays an essential role in maintaining oral health. In addition to a large amount of water, saliva is also composed of a variety of electrolytes, protein components and numerous volatile organic compounds, some of which are produced by microorganisms in the oral cavity, such as aliphatic amines, branched chain fatty acids, indole, phenol, and volatile sulfur compounds (Cheng et al., 2016). External environmental stimuli may lead to changes in the oral microflora, thereby changing the amount of volatile organic compounds in saliva. The loss of salivary gland function can also cause changes in oral microorganisms, resulting in alterations in oral microflora, thus changing the caries process.

At present, studies on microbial diversity of caries are mostly conducted at the gene level. It is well known that genomics explores life activities at the gene level, but in fact, many life activities in cells occur at the metabolite level, cellular signal releasing, energy transfer and intercellular communication are regulated by metabolites. Untargeted metabolomics can identify differentially expressed metabolites through univariate and multivariate analysis methods, thereby reflecting the environment in which the cell is located, and the interrelationship between the cellular environment and external influences. Goldsmith et al. (Goldsmith et al., 2010) believe that metabolomics plays an important role in disease diagnosis in clinical practice. Moreover, basic research on saliva has received much attention in recent years, saliva has obvious advantages in the early diagnosis of diseases (Cheng et al., 2014; Zhang et al., 2016). Broad-spectrum metabolomics analysis is a research technique that can quantitatively analyze all metabolites in organisms, and find the relative relationship between metabolites and physiological and pathological changes. The research objects in metabolomics are small molecular substances with a relative molecular mass weight of less than 1000 Da, such as lipids, ketones, and organic acids.

About 60%-90% of children in the world are affected by caries(Petersen et al., 2005; Listl et al., 2015). The oral cavity of children with mixed dentition has a special local environment, which determines its different microbial characteristics(Xu et al., 2018; Yang, 2020). Saliva is a key component in the defense against microbial attack and can reflect various biological information of the whole caries and pathogenic microorganisms (Zhang et al., 2014). Saliva metabolomics mainly targets small molecules from dysfunctional microbiome and host tissue destruction. Saliva metabolic profile can reflect the molecular phenotype of oral health in real time. People pay more and more attention to the identification of metabolites from saliva and other oral fluids (Mikkonen et al., 2016; Buzalaf et al., 2020; Kim et al., 2021). At present, there are few studies on mixed dental caries, only a few scattered reports, and the related studies need to be further developed.

Therefore, in this study, we performed untargeted metabolomics *via* liquid chromatography tandem mass spectrometry (LC-MS/MS) on saliva samples obtained from caries-active and caries-free children in the mixed dentition, and explored the differences in salivary metabolites between caries-active and caries-free children, so as to identify possible key metabolites and related pathways, and provide new ideas for the prevention and treatment of caries.

## 2 Materials and methods

### 2.1 Subjects

A total of 20 children aged 8-9 years in the mixed dentition who received Children’s oral health examination in Yubei District, Chongqing city in December 2021 were recruited. There were 10 caries-active (aged 8.6 ± 0.49years) and 10 caries-free children(aged 8.5 ± 0.5years), the male/female ratio was 1:1. Written informed consent was obtained from the parent or guardian of the children.

### 2.2 Inclusion criteria

Inclusion criteria for the caries-active group included: children who had the same number of permanent and deciduous teeth in the oral cavity, no systemic disease or congenital disease, no developmental anomaly, and no bacterial or serious infections in other parts of the body; children who didn’t take antibiotics, fluoride and microecological modulators, and wore removable oral dental appliances within the last 3 months; there is no redness, swelling and bleeding in the gums.DMFT and dmft index were used to record caries. When the number of caries was more than 6, caries were considered as high caries group. In order to eliminate the interference of M and F as much as possible of the oral environment, and only D and d were counted in the mouth. Meanwhile, to find out whether children with high caries and children without caries have different metabolites in saliva involved in caries progression through a small number of samples, this study increased the number of caries samples, that is, the total number of D and d counts more than 10 teeth to be considered for inclusion.

Inclusion criteria for the caries-free group included: children who had the same number of permanent and deciduous teeth in the oral cavity, no systemic disease or congenital disease, no developmental anomaly, and no bacterial or serious infections in other parts of the body; children who didn’t take antibiotics, fluoride and microecological modulators, and wore removable oral dental appliances within the last 3 months; children who had no caries or fillings in either a primary or permanent tooth.And there is no redness, swelling and bleeding in the gums.

### 2.3 Saliva sample collection

According to Rhodus’ modified method (Rhodus et al., 2005), whole non-stimulated saliva was collected from all participants. The collection time was between 9:00 am to 11:00 am, with a 1-hour fasting before saliva collection. During collection, all participants were asked to keep the saliva in the mouth for at least 1min, and then spit whole saliva, without cough up mucus, into a centrifuge tube/sterile container. To ensure that enough saliva (2-5mL) was collected, the process may need to be repeated several times. The obtained saliva samples were placed on ice, transported to the laboratory immediately, and centrifuged at 5000g at 4°C for 10min. The supernatant was collected, and sterilized by filtration through 0.22 µm filters. 1mL of saliva samples was aliquoted into labeled 2 mL Eppendorf tubes, and stored in the -80°C freezer. Saliva samples were taken and thawed before untargeted metabolomics analysis. In order to avoid errors caused by operator in the collection process, all samples were completed by one operator.LC-MS/MS analysis, data analysis and processing was carried out at Huada Medical Laboratory Co., Ltd. (Wuhan, China).

Extraction and preparation steps of saliva metabolites:①Add 100µL of each sample into the corresponding centrifuge tube, and freeze the remaining samples;②Add 700 µ L of extractant containing internal standard 1 (methanol: acetonitrile: water=4:2:1), shake for 10min, and place it in a refrigerator at - 20 °C for 2h;③25000g and centrifuge at 4°C for 15min;④The sample is removed from the centrifuge and 600µL of supernatant is transferred to a new centrifuge tube;⑤Drain with a drainer;⑥Add 180µL methanol: pure water (1:1 v/v) and swirl for 10min until completely dissolved in the complex solution;⑦25000g and centrifuge at 4°C for 15min again;⑧The remaining samples were taken 50µL each into the three upper plates for the detection of positive and negative ions, and the other plate was used as the spare plate, and the remaining samples were taken 20µL mixed QC.

### 2.4 Untargeted metabolomics analysis of saliva samples

LC-MS/MS technology was used to perform untargeted metabolomics on saliva collected from caries-active and caries-free children. High-resolution LC-MS/MS mass spectrometer Q Exactive (Thermo Fisher Scientific, USA) was used to acquire data in the positive and negative ion modes, thus improving metabolite coverage. LC-MS/MS data processing was performed using Compound Discoverer 3.1 software (Thermo Fisher Scientific, USA), mainly for peak picking, peak alignment and identification of compounds.

#### 2.4.1 Chromatographic conditions

The samples were analyzed on a Waters 2D UPLC (Waters, USA), coupled to a Q-Exactive mass spectrometer (Thermo Fisher Scientifific, USA) with a heated electrospray ionization (HESI) source and controlled by the Xcalibur 2.3 software program (Thermo Fisher Scientifific, Waltham, MA, USA). Chromatographic separation was performed on a Waters ACQUITY UPLC BEH C18 column (1.7 μm, 2.1 mm × 100 mm, Waters, USA), and the column temperature was maintained at 45°C. The mobile phase consisted of 0.1% formic acid (A) and acetonitrile (B) in the positive mode, and in the negative mode, the mobile phase consisted of 10 mM ammonium formate (A) and acetonitrile (B). The gradient conditions were as follows: 0-1 min, 2% B; 1-9 min, 2%-98% B; 9-12 min, 98% B; 12-12.1 min, 98% B to 2% B; and 12.1-15min, 2% B. The flow rate was 0.35 mL/min and the injection volume was 5 μL.

#### 2.4.2 Mass spectrometry conditions

The mass spectrometric settings for positive/negative ionization modes were as follows:spray voltage, 3.8/−3.2 kV; sheath gas flow rate, 40 arbitrary units (arb); aux gas flflow rate, 10 arb; aux gas heater temperature, 350°C; capillary temperature, 320°C. The full scan range was 70–1050 m/z with a resolution of 70000, and the automatic gain control (AGC) target for MS acquisitions was set to 3e6 with a maximum ion injection time of 100 ms. Top 3 precursors were selected for subsequent MS/MS fragmentation with a maximum ion injection time of 50 ms and resolution of 17500, the AGC was 1e5. The stepped normalized collision energy was set to 20, 40 and 60 eV.

In order to provide more reliable experimental results during instrument testing, the samples are randomly ordered to reduce system errors. A QC sample is interspersed for every 10 samples.

### 2.5 Statistical analysis

R software package metaX was used for data preprocessing, statistical analysis, classification and functional annotation of metabolites (Wen et al., 2017). Principal component analysis (PCA) was used for dimensionality reduction of multivariate original variables in order to detect the groups, trends (similarities and differences within and between grouped samples) and outliers (the presence and absence of outlier samples) of the observed variables in the dataset. The combination of the variable influence in projection (VIP) scores of the first two principal components of the partial least squares-discriminant analysis (PLS-DA) model(Barker and Rayens, 2003; Westerhuis et al., 2008), the fold-changes from the univariate analysis, and Student’s t test was used to screen differential metabolites.

In a strict sense, biological data did not strictly obey normal distribution. Before T test, we processed the data by log2 to make the data approximate obey normal distribution, so the result of T test is relatively more reasonable.At the same time, considering that the difference between groups is not so significant, we used p-value(Zheng et al., 2019) as the condition for screening the difference in order to screen the appropriate differential metabolites for subsequent research, and did not correct it.

## 3 Results from salivary metabolomics

### 3.1 Quality control of samples

**Figure 1:**
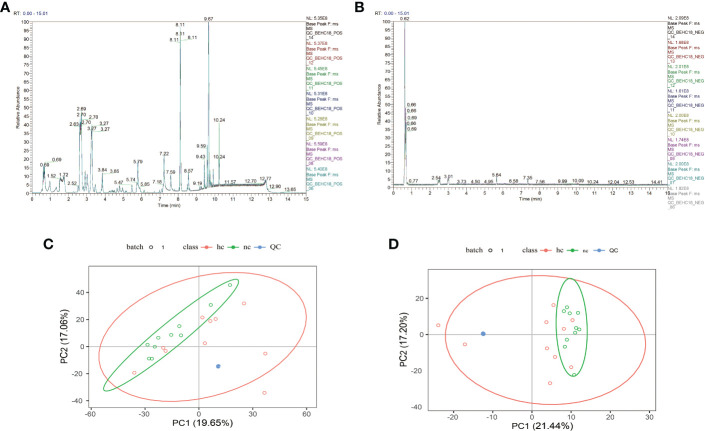
**(A, C)**: Positive ion mode; **(B, D)**: Negative ion mode.

As shown in **Figures 1A, B**, after overlapping the base peak ion chromatograms of all quality control samples, the chromatograms well overlapped in both the positive and negative ion modes, the retention time and peak response intensity fluctuated little, indicating that the instrument was in a good state with stable signal during the whole sample detection process.A PCA analysis of the QC sample and all samples can be used to observe the overall distribution of each set of samples and the stability of the entire analytical process. As shown in **Figures 1C, D**, the better the QC samples aggregate, the more stable the instrument and the better the repeatability of the acquired data.

### 3.2 Compound detection results

The results of the present study showed that in the positive ion mode, a total of 1606 molecular features were detected in the saliva samples of the two groups, 543 of which could be identified using the Chemspider and mzCloud databases with corresponding compound information. In the negative ion mode, a total of 532 molecular features were detected, 204 of which could be identified using the Chemspider and mzCloud databases with corresponding compound information (**Table 1**).

**Table 1:** Number of compounds and number of compounds with identification information detected in both the positive and negative ion modes.

Mode	Number of compounds	Number of compounds with identification information
Positive ion mode (pos)	1606	543
Negative ion mode (neg)	532	204

Positive ion mode (pos): when the substances are ionized in an ion source, the adduct ions are positive ions, such as H^+^, ${\text{NH}}_{4}^{+}$ , Na^+^, K^+^.

Negative ion mode (neg): when the substances are ionized in an ion source, the adduct ions are negative ions, such as -H, +Cl.

### 3.3 Classification of the identified metabolites

**Figure 2:**
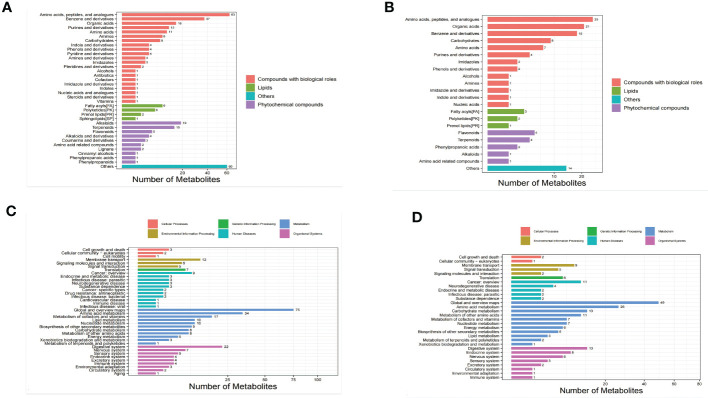
Bar chart of metabolite classification in the positive ion mode **(A)** and negative ion mode **(B)**. X-axis represents the number of metabolites in each class, Y-axis represents the metabolite classification entries. Bar chart of KEGG functional annotation of metabolites in the positive ion mode **(C)** and negative ion mode **(D)**.

The identified metabolites were classified using the Kyoto Encyclopedia of Genes and Genomes (KEGG) database and the Human Metabolome Database (HMDB) in order to understand the classification of metabolites. The number of metabolites in each class is shown in **Figures 2A, B**. Others means that classification information is the remaining categories. Identification results without classification information were not included in the analysis. At the same time,the identified metabolites were functionally annotated by the KEGG database in order to understand their functional properties, and determine the major biochemical metabolic pathways and signal transduction pathways involved in the metabolites. The number of metabolites identified in each type of metabolic pathways is shown **Figures 2C, D**.

The results showed that among the 543 molecular features identified in the positive ion mode, 315 molecular features were classified into four categories, including compounds with biological roles (n = 184), lipids (n=18), phytochemical compounds (n = 53), and others (n = 60). Among the 204 metabolites identified in the negative ion mode, 126 molecular features were classified into four categories, including compounds with biological roles (n = 93), lipids (n=6), phytochemical compounds (n = 13), and others (n = 14) (**Table 2**).

**Table 2:** Classification and number of metabolites detected in the positive and negative ion modes.

Classification Mode	Compounds with biological roles	Lipids	Phytochemical compounds	Others
Positive ion mode	184	18	53	60
Negative ion mode	93	6	13	14

Bar chart of KEGG functional annotation of metabolites in the positive ion mode (2C) and negative ion mode (2D). X-axis represents the number of metabolites in the pathway, and Y-axis represents KEGG pathway entries.

The results showed that in the positive ion mode, 38 KEGG pathways containing 298 metabolites were annotated, and the top 4 pathways, sorted in descending order by the number of metabolites in each pathway were as follows: global and overview maps (75 metabolites), amino acid metabolism (34 metabolites), digestive system (22 metabolites), and metabolism of cofactors and vitamins (17 metabolites). The number of metabolites contained in these 4 pathways accounted for 49.67% of all metabolites annotated to the pathways. In the negative ion mode, 30 KEGG pathways containing 211 metabolites were annotated, and the top 4 pathways, sorted in descending order by the number of metabolites in each pathway were as follows: global and overview maps (49 metabolites), amino acid metabolism (26 metabolites), digestive system (13 metabolites), and carbohydrate metabolism (13 metabolites). The number of metabolites contained in these 4 pathways accounted for 47.87% of all metabolites annotated to the pathways. These findings suggested that the metabolites with identification information detected and identified in either positive or negative ion mode functioned mainly through two types of pathways, metabolism and organismal systems.

### 3.4 Statistical analysis results

**Figure 3:**
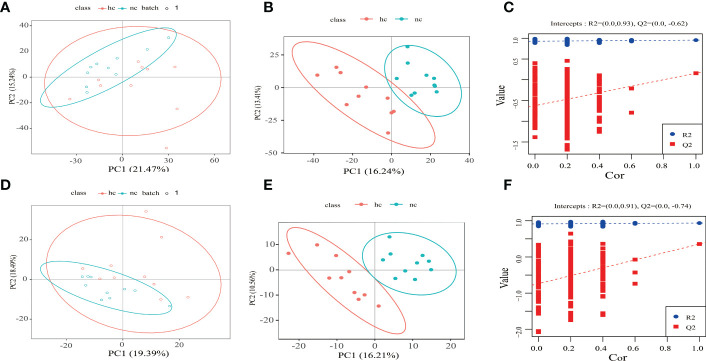
**(A–C)** Positive ion mode; **(D–F)** Negative ion mode. hc: high caries; nc: no caries. **(A, D)** PCA results The abscissa is the first principal component PC1, the ordinate is the second principal component PC2, and the ellipse in the PCA score graph is 95% confidence interval. Each dot represents a sample, and different groups are labeled with different colors. hc: high caries; nc: no caries. The number is the score of the principal component, which represents the percentage of the explanation on overall variance of the specific principal component. **(B, E)** PLS-DA results. The horizontal axis is the first principal component, and the vertical axis is the second principal component. The number in brackets is the score of the principal component, indicating the percentage of total variance explained by the corresponding principal component. **(C, F)** The two rightmost points in the figure are the actual R2Yand Q2 values of PLS-DA model, and the remaining points are the R2Y and Q2 values obtained by randomly arranging the samples used.

Based on the following conditions: 1) the VIP of the first two principal components of the PLS-DA model ≥ 1; 2) fold-change ≥ 1.2 or ≤ 0.83; and 3) P-value< 0.05, the differential metabolites between caries-active group and the caries-free group was identified (**Figure 3**).

By comparing the caries-active group with the caries-free group, a total of 189 differential metabolites were identifiedin the positive ion mode, including104 up-regulated metabolites, and 85 down-regulated metabolites. A total of 70 differential metabolites were identified in the negative ion mode, including 37 up-regulated metabolites, and 33 down-regulated metabolites (**Table 3**).

**Table 3:** Differential metabolites identified in the positive and negative ion modes in the caries-active group compared to the caries-free group.

Differential metabolites Mode	Total differential metabolites identified	Up-regulated metabolites	Down-regulated metabolites
Positive ion mode	189	104	85
Negative ion mode	70	37	33

### 3.5 Results from volcano plot and metabolic pathway enrichment analysis of differential metabolites

**Figure 4:**
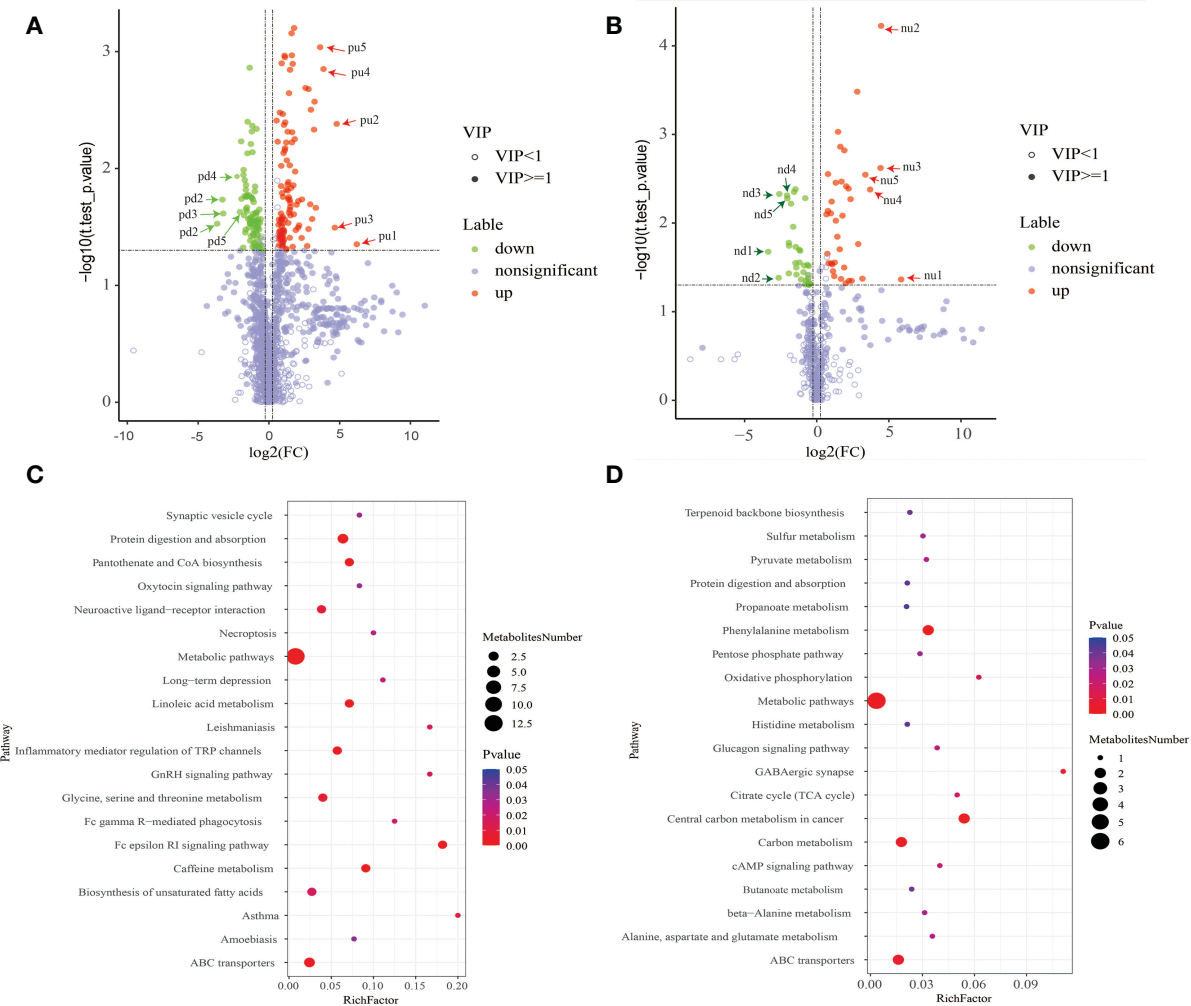
**(A, C)** The positive ion mode; **(B, D)** the negative ion mode. **(A, B)** Results from volcano plot analysis.Blue indicates significantly down-regulated differential metabolites, and red indicated significantly up-regulated differential metabolites, purple-gray indicates metabolites with no significant difference. **(C, D)** Results of metabolic pathway enrichment analysis of differential metabolites. Metabolic pathways with a p-value< 0.05 were significantly enriched by differential metabolites. The X-axis represents the rich factor, the greater the rich factor, the greater the ratio of different metabolites annotated to this pathway. The dot size represents the number of differential metabolites annotated to this pathway. Enrichment analysis was based on annotated metabolites in KEGG database. The annotation results of differentiated metabolites screened in this project were statistically analyzed by combining hypergeometric test, and the p-value of corresponding pathway was obtained. Then, p-value<0.05 was taken as the threshold to determine whether the pathway was enriched or not. The ggplot2 package in the R package is used for mapping.

The results from metabolic pathway enrichment analysis of differential metabolites showed that in the positive ion mode, the top 3 enriched metabolic pathways that were significantly altered between two groups included Asthma (the 27th most up-regulated metabolite, histamine was enriched in this pathway), Fc epsilon RI signaling pathway (the 27th and 46th most up-regulated metabolites, histamine and arachidonic acid were enriched), and GnRH signaling pathway (the 46th most up-regulated metabolite, arachidonic acid was enriched). In negative ion mode, the top 3 significantly altered metabolic pathways were GABAergic synapse (the 13th up-regulated metabolite, succinate was enriched), oxidative phosphorylation (the 13th up-regulated metabolite, succinate was enriched), and central carbon metabolism in cancer (the 13th and 37th most up-regulated metabolites, succinate and L-histidine were enriched). In contrast, the changes in the Metabolism pathway that was enriched by the top-ranking up-regulated differential metabolites were the smallest among the significantly altered metabolic pathways (**Figure 4**).

As shown in **Table 4**, among the top 5 up-regulated differential metabolites, only one metabolite named Theobromine (molecular formula C7H8N4O2) that had compounds with biological roles, and was classified as purines and derivatives could be annotated to caffeine metabolism (pathway ID, 00232, sub-class: Biosynthesis of other secondary metabolites, class: Metabolism), and metabolic pathways (pathway ID, 01100, sub-class: Global and overview maps, class: Metabolism). Additionally, Daidzein (molecular formula C15H10 O4) that was classified as flavonoids, and Flazin (molecular formula C17H12N2O4) that was classified as alkaloids and derivatives, were phytochemical compounds, but these two metabolites were not annotated to specific pathways. Among the top 5 down-regulated differential metabolites, only one metabolite named hostmaniane (molecular formula C13H18O5) was classified as benzene and derivatives, which had compounds with biological roles, but was not annotated to specific pathways.

**Table 4:** Detailed information about top 5 (pu1-pu5) up-regulated and top 5 (pd1-pd5) down-regulated differential metabolites in the positive ion mode.

	Name	ChemSpider ID mzCloud	Molecular Formula	Molecular.Weight	KEGG.ID	Pathway	Level	Family	Metabolites
pu1	(2s,8r,15e)-1,2,5,14-tetrahydroxy-8-{[(9z)-9-octadecenoyloxy]methyl}-10-oxo-4,6,9-trioxa-5-phosphaheptadec-15-en-17-oic acid 5-oxide	113380983	C_32_H_57_O_13_P	680.3548Da	–	–	Level 4	–	–
pu2	Flazin	4526683	C_17_H_12_N_2_O_4_	308.0797Da	–	–	Level 4	Alkaloids and derivatives	Phytochemical compounds
pu3	Theobromine	Reference-676	C_7_H_8_N_4_O_2_	180.065Da	C07480	2	Level 1	Purines and derivatives	Compounds with biological roles
pu4	Daidzein	Reference-680	C_15_H_10_ O_4_	254.058Da	C10208	–	Level 2	Flavonoids	Phytochemical compounds
pu5	–	–	C_20_H_43_O_4_P	378.2883Da	–	–	Level 5	–	–
pd1	–	–	C_8_ H_19_ NOS	177.1196Da	–	–	Level 5	–	–
pd2	–	–	–	217.1152Da	–	–	Level 5	–	–
pd3	–	–	C_10_ H_23_ NO_2_ P_2_	251.1209Da	–	–	Level 5	–	–
pd4	Hostmaniane	9359446	C_13_H_18_O_5_	254.1155Da	–	–	Level 4	Benzene and derivatives	Compounds with biological roles
pd5	–	–	–	458.2367Da	–	–	Level 5	–	–

As shown in **Table 5**, two metabolites named Daidzein (molecular formula C15H10 O4) and Genistein (molecular formula C15H10 O5) were phytochemical compounds, and both classified as flavonoids. Genistein was annotated to metabolic pathways (pathway ID, 01100, class: Metabolism, sub-class: Global and overview maps). Among the top 5 down-regulated metabolites in the negative ion mode, only one metabolite named N-[(4-methoxy-1-benzofuran-5-yl)carbonyl]glycine (molecular formula C12 H11 NO5) was classified as amino acids, peptides, and analogues, and was compounds with biological roles, but the specific pathways were not identified.

**Table 5:** Detailed information about top 5 (pu1-pu5) up-regulated and top 5 (pd1-pd5) down-regulated differential metabolites in Negative ion mode.

	Name	ChemSpider ID mzCloud	Molecular Formula	Molecular.Weight	KEGG.ID	Pathway	Level	Family	Metabolites
nu1	–	–	C_13_H_24_N_2_O_4_	272.1732Da	–	–	Level 5	–	–
nu2	Daidzein	Reference-680	C_15_H_10_O_4_	254.0575Da	C10208	–	Level 2	Flavonoids	Phytochemical compounds
nu3	Genistein	Reference-24	C_15_H_10_O_5_	270.0523Da	C06563	1	Level 2	Flavonoids	Phytochemical compounds
nu4	–	–	–	188.0491Da	–	–	Level 5	–	–
nu5	–	–	C_19_H_32_N_6_O_9_P_2_	550.1732Da	–	–	Level 5	–	–
nd1	–	–	C_8_H_19_NOS	177.1191Da	–	–	Level 5	–	–
nd2	similar to: argininosuccinic acid;δmass: -73.0079 da	–	–	217.1147Da	–	–	Level 5	–	–
nd3	–	–	C_44_H_72_N_6_O_18_	972.4932Da	–	–	Level 5	–	–
nd4	–	–	–	147.9732Da	–	–	Level 5	–	–
nd5	N-[(4-methoxy-1-benzofuran-5-yl)carbonyl]glycine	74852585	C_12_ H_11_ NO_5_	249.0633Da	–	–	Level 4	Amino acids, peptides, and analogues	Compounds with biological roles

## 4 Discussion

Based on our previous research on the microbial diversity of children with high caries and children without caries, the cumulative species curve at the species and genus levels showed that when the sample size reached 10 cases, the new species did not increase significantly with the increase of the sample size in the different environment with high caries and no caries. At the same time, the occurrence of common species tends to be saturated. Therefore, we can try to compare the saliva metabolism of children with extreme high caries and those without caries, to find out the different metabolites related to caries, which used for only 10 samples in each group of this study.

Matching of three dimensions retention time, MS1 and MS2 spectra with standard spectral libraries is currently the most widely used reliable approach for metabolite identification in metabolomics (Liang et al., 2020; Shen et al., 2020). In terms of confidence levels of metabolites identified in the present study, among the top 5 up-regulated and top 5 down-regulated differential metabolites identified in both the positive and negative ion modes (20 metabolites), only 4 were identified at level 2 or higher, the remaining metabolites were identified at level 4 or lower. The results indicated that although a variety of differential metabolites were identified, but relatively few metabolites with high confidence were available for further analysis, and their biological information needs to be further explored and analyzed.

Arachidonic acid is a polyunsaturated fatty acid that is released in large amounts by macrophages (Rouzer et al., 2006)and neutrophilic macrophages (Walsh et al., 1981) during the inflammatory burst. Beavers et al. revealed that arachidonic acid could kill *Staphylococcus aureus* through a lipid peroxidation mechanism (Beavers et al., 2019). A study on arachidonic acid and its metabolites in middle ear effusion of secretory otitis media, and bacterial culture of middle ear effusion showed that there was no statistically significant correlation between bacterial culture results (positive or negative) and concentrations of arachidonic acid and its metabolites in middle ear effusion (Chen, 2018). At present, for oral diseases, arachidonic acid and its metabolites are mostly investigated in periodontitis (Luo and He, 2015)and oral squamous carcinoma (Han, 2016), however, there is a lack of studies concerning their correlation with caries. Although our results showed that arachidonic acid was a metabolite that significantly up-regulated in the caries-active group compared with the caries-free group, and the pathway that was enriched by this metabolite was the top-ranked pathway, but it is questionable whether it can be a new direction for caries research.

Histamine, an organic nitrogenous compound, is generated by decarboxylation of the histidine. Salivary histatins are a family of histidine-rich cationic homologous polypeptides with similar amino acid sequences, which are considered to be important components of non-immune host defense system in the oral cavity. Studies have confirmed that the concentrations of slivary histatin-5 was associated with the severity of caries, histatin-5 concentrations in saliva of children without caries was significantly higher than that of children with caries, which decreased gradually with the increase in caries severity (Munther, 2020). Therefore, it is believed that salivary histatin-5 can be applied in the prevention and treatment of caries, especially in children with caries [Jurczak et al., 2015; Munther, 2020). Krzysciak et al. (Krzysciak et al., 2015)showed that salivary histatin-5 had the ability to inhibit the growth of *Streptococcus mutans in vitro* and the dental plaque biofilm formation (Krzysciak et al., 2015). Huo et al. reached a similar conclusion, and also confirmed that histatin-5 could inhibit the coaggregation of cells of *Streptococcus mutans* with other species of microbiota, thus reducing the adhesion of *Streptococcus mutans* on the teeth and gums surface (Huo et al., 2011). Fernández-Presas et al. (Fernández-Presas et al., 2018)used transmission electron microscopy to observe the ultrastructure of *Streptococcus mutans*, and found that salivary histatin-5 could penetrate the cell membranes and cell walls of *Streptococcus mutans*, accumulate in the bacterial cytoplasm, interact with bacterial DNA, thus leading to cell death. Other previous studies have found that salivary histatin-5 could be attracted to the teeth enamel surface through electrostatic attraction, effectively inhibiting enamel demineralization, resisting the breakdown of teeth due to acids produced by bacteria to a certain extent (Siqueira et al., 2010; Zhou et al., 2020). In the present study, results from the metabolic pathway enrichment analysis of differential metabolites showed that the expression of histamine and histidine were significantly up-regulated in the caries-active group compared to the caries-free group. It can be hypothesized that the increase in histamine may be due to the increase in histidine, and the increase in histidine my be caused by the enhanced hydrolysis of histatins, since histatins is a histidine-rich homologous polypeptide. Our results are consistent with the findings from a study of Munther et al, which revealed decreased histatin-4 concentrations in the saliva of caries-active children (Munther, 2020). However, further studies are needed to confirm the aforementioned hypothesis, i.e. enhanced hydrolysis of histatins may lead to an increase in histidine, which in turn leads to an increase in histamine under caries-active environment.

The succinate dehydrogenase (SDH) catalyzes the oxidation of succinate to fumarate in the tricarboxylic acid cycle (TCA). Early studies found that when caries involved the dentin, the surrounding dentinal tubules were strongly positive for SDH (Lisanti, 1961; Wu, 1979), and these positive areas were distributed in the sites of bacteria accumulation, such as plaque, infected dentinal tubules, exhibiting spherical, and filamentous morphologies, which was consistent with bacterial morphology, indicating that the bacteria at the caries lesions had strong enzymatic activity (Wu, 1979). The results of this present study showed that the expression of succinate was significantly up-regulated in the caries-active group compare to the caries-free group, this result seems to confirm the changes in SDH activity in the saliva of caries-active childrens indirectly. On the one hand, succinate accumulation is associated with impaired SDH activity due to hypoxia (Wayne and Lin, 1982; Cimen et al., 2010; He et al., 2012); on the other hand, reversible conversion of glutamate to α-ketoglutarate by glutamate dehydrogenase, and increased γ-aminobutyric acid transport can also lead to succinate accumulation (Mills and O’Neill, 2014). SDH can not only catalyze TCA cycle, but also act as electron transport chain. When SDH acts as an electron transfer chain and is involved in oxidative phosphorylation, it transfers electrons from succinate to ubiquinone through the FeS cluster(Miyadera et al., 2003). Under hypoxic conditions, inhibition of hypoxia-induced succinate accumulation can lead to cell death, indicating that succinate metabolism is important for cell survival (Eoh and Rhee, 2013). In the present study, we found that in the negative ion mode, the top 2 most significantly-altered metabolic pathways were GABAergic synapse and Oxidative phosphorylations. The results are also consistent with the findings of the above-mentioned studies.

A study on bacterial composition and metabolomics of dental plaque from adolescents conducted by Havsed et al. (Kristian et al., 2021) showed that for adolescents with caries, several organic acids (acetate, succinate, and lactate) formed a cluster that was highly positively correlated with *Streptococcus constellatus, Scardovia wiggsiae, Capnocytophaga ochracea, Veillonella tobetsuensis, Atopobium parvulum, or Actinomyces sp*, however these organic acids did not appear to be significantly correlated with any species in the caries-free group. Correlation analysis in the caries-free group revealed positive correlation between oral microbes associated with oral health such as *S. oralis, S. parasanguinis, Corynebacterium durum, Rothia dentocariosa or R. mucilaginosa* and succinate. Although bacterial correlation analysis was not performed in the present study, but the results of the present study revealed significantly up-regulated succinate in saliva of caries-active children compared to caries-free children, which are similar to the findings of Havsed et al. The available data indicate that a correlation may exist between succinate are and caries, but further studies are needed to investigate the role of succinate in bacterial biofilm formation, and the signal transduction between bacteria and the host during caries process. The changes in SDH levels and activity during caries process also require further investigation.

## 5 Conclusions

**Figure 5:**
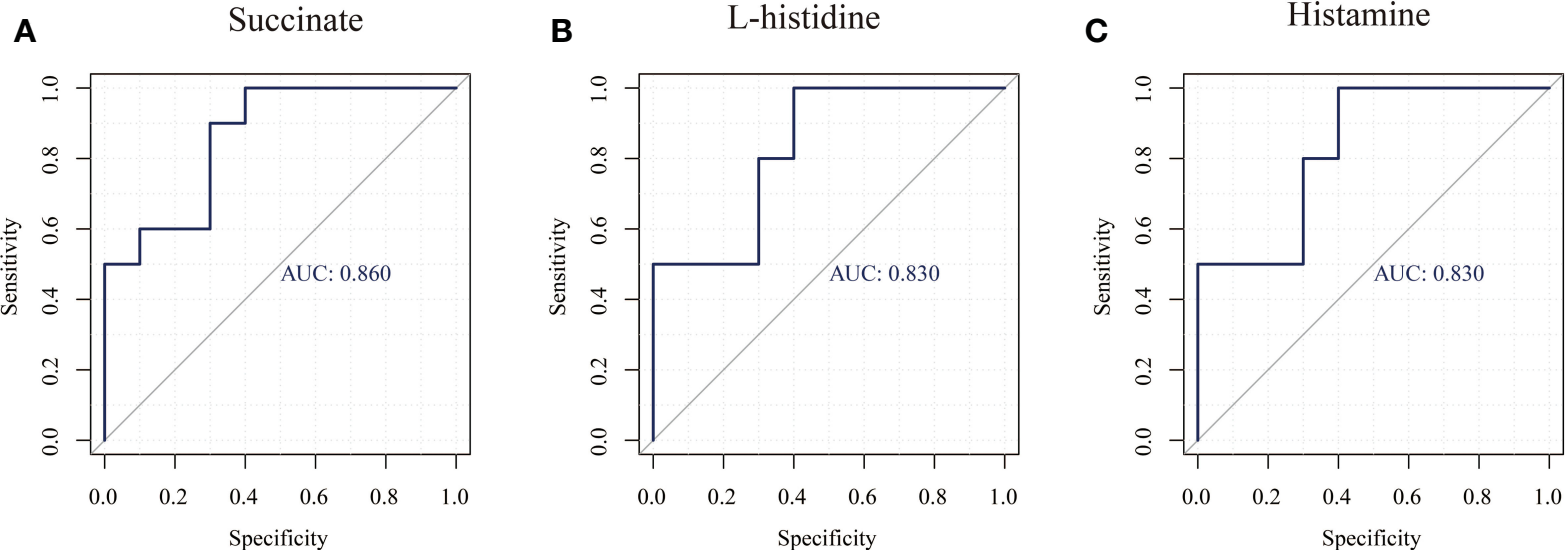
ROC curves of three major differential metabolites (Succinate, Histamine, L-histidine). **(A)** Succinate, **(B)** Histamine, **(C)** L-histidinet.

In this study, untargeted metabolomics analysis of the saliva from caries-active and caries-free children in mixed dentition was carried out by LC-MS/MS, our findings confirmed that the enriched differential metabolites histamine, L-histidine, succinate were related to the presence of caries (**Table 6** and **Figure 5**), but their role in the caries process remains to be further studied.

**Table 6:** Correlation information of four major differential metabolites in pathway enrichment analysis.

Name	Compound.ID	Actual.RT	ChemSpider ID mzCloud	Molecular Formula	Molecular.Weight	KEGG.ID	Pathway	Level	Family	Metabolites
Histamine	0.569_111.0799	0.569	S753	C_5_H_9_N_3_	111.0799Da	C00388	9	Level 4	Amines	Compounds with biological roles
L-histidine	0.698_155.0694	0.698	Reference-473	C_6_H_9_N_3_O_2_	155.0694Da	C00135	8	Level 2	Amino acids	Compounds with biological roles
Succinate	0.64_118.0267	0.64	BGI525	C_4_H_6_O_4_	118.0267Da	C00042	18	Level 1	Organic acids	Compounds with biological roles
Arachidonic								Level 2		Lipids
acid	9.908_304.2398	9.908	Reference-2742	C_20_H_32_O_2_	304.2398Da	C00219	21		Fatty acyls	

## Data availability statement

The raw data supporting the conclusions of this article will be made available by the authors, without undue reservation.

## Ethics statement

The studies involving human participants were reviewed and approved by Research Ethical Committee, The Affiliated Hospital of Stomatological, Chongqing Medical University. Written informed consent to participate in this study was provided by the participants’ legal guardian/next of kin. Written informed consent was obtained from the individual(s), and minor(s)’ legal guardian/next of kin, for the publication of any potentially identifiable images or data included in this article.

## Author contributions

YL, ZY, TC conceived the research theme and supervised the entire study. YL collected the data, analyzed the data, drew the figures, explained the results, and drafted the manuscript. DJ, JL and ZZ revised the manuscript and performed reference collection. All authors contributed to the article and approved the submitted version.
